# Supplementary material for: Inequality of household consumption and air pollution-related deaths in China
Source: Nat Commun. 2019 Sep 25;10:4337. doi: 10.1038/s41467-019-12254-x (PMC6761204; doi:10.1038/s41467-019-12254-x)
Supplement: Supplementary file 1 — Supplementary Information [file 41467_2019_12254_MOESM1_ESM.pdf]

Supplementary Information for

Inequality of household consumption and air pollution-related deaths in China

Zhao et al.

Table of Contents

Supplementary Figures 3

Supplementary Figure 1. 3

Supplementary Figure 2. 4

Supplementary Figure 3. 6

Supplementary Figure 4. 7

Supplementary Figure 5. 8

Supplementary Figure 6. 9

Supplementary Figure 7. 5

Supplementary Tables 10

Supplementary Table 1 10

Supplementary Table 2. 11

Supplementary Table 3. 12

Supplementary Table 4. 14

Supplementary Table 5. 15

Supplementary Discussion 16

Supplementary References 18

Supplementary Figures

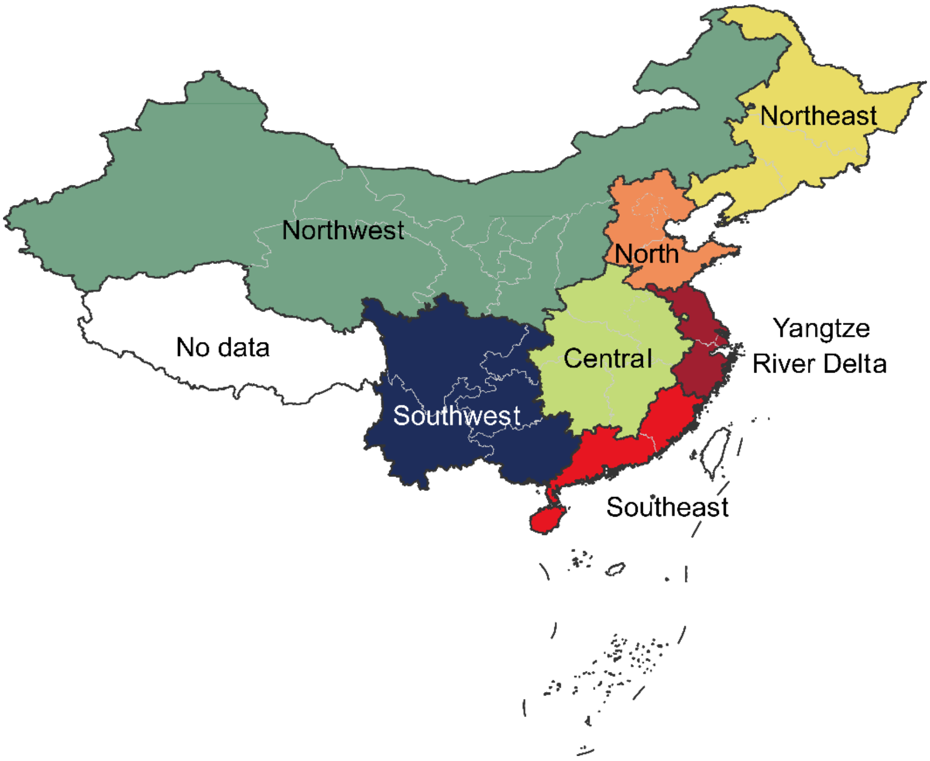

Supplementary Figure 1. Region definitions.

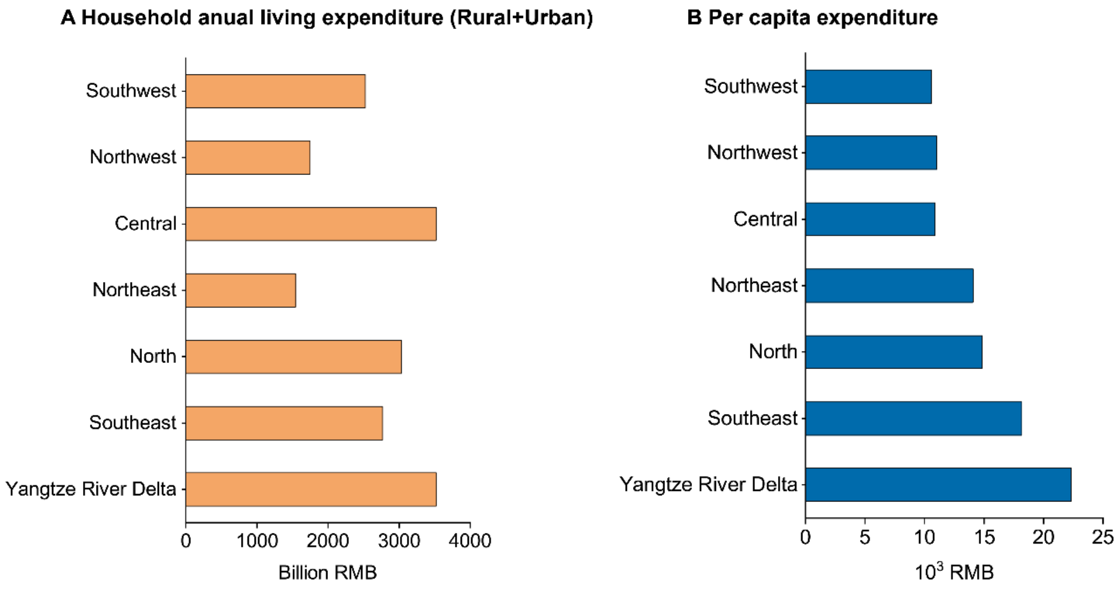

Supplementary Figure 2. Household (A) annual total and (B) per capita living expenditure for the seven regions. Source data are provided as a Source Data file.

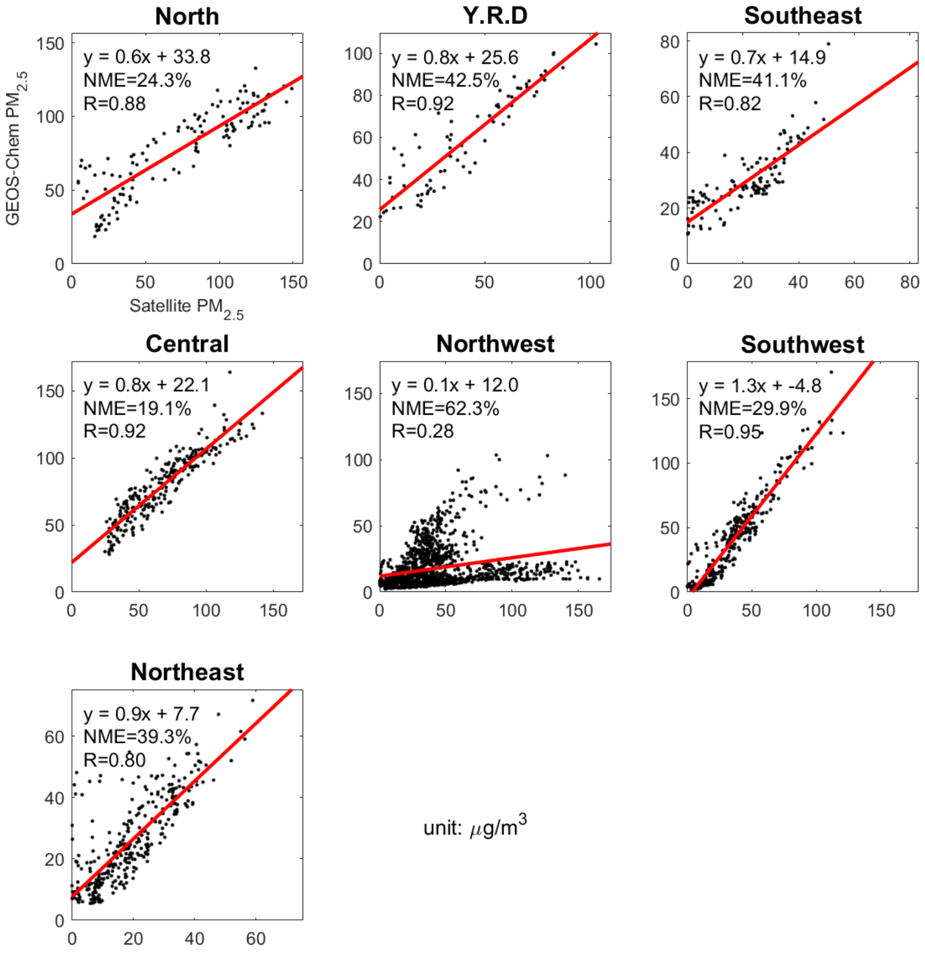

Supplementary Figure 3. Comparisons between the simulated and satellite-derived $PM_{2.5}$ concentrations over the seven regions in 2012.

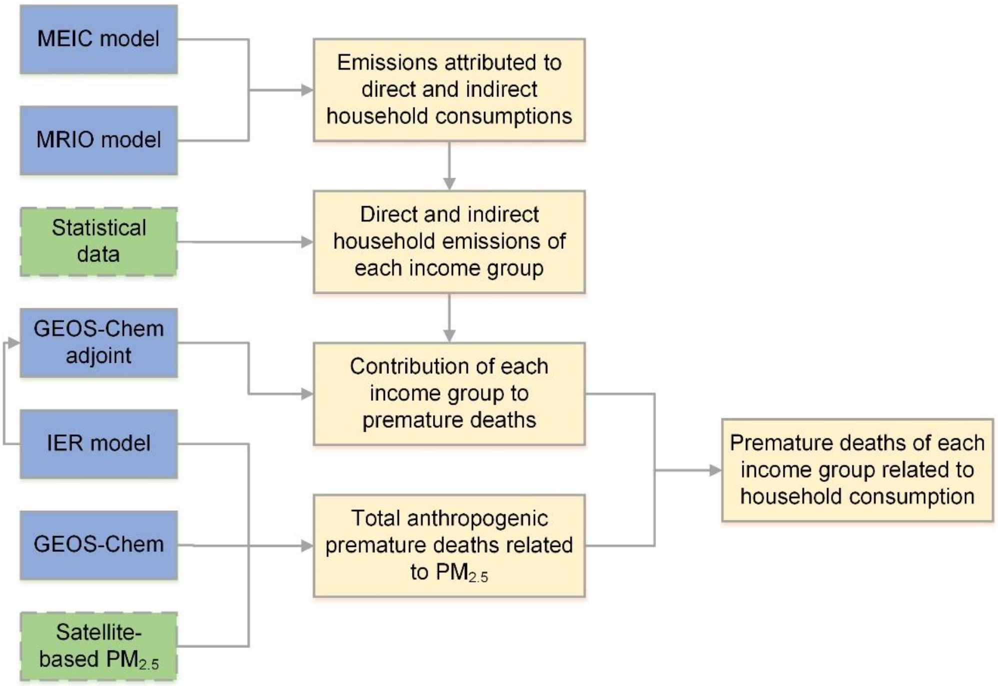

Supplementary Figure 4. Schematic of the methodology used in this study.

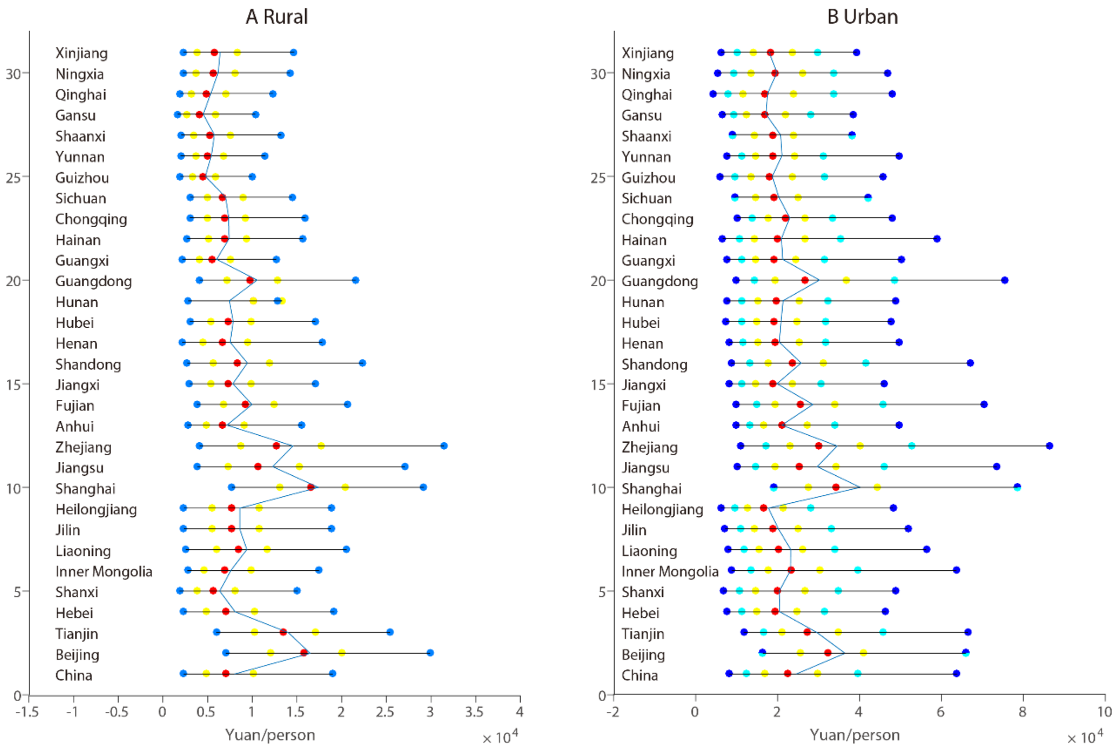

Supplementary Figure 5. Household income per capita of each group in (A) rural and (B) urban areas of 30 provinces in 2012. Source data are provided as a Source Data file.

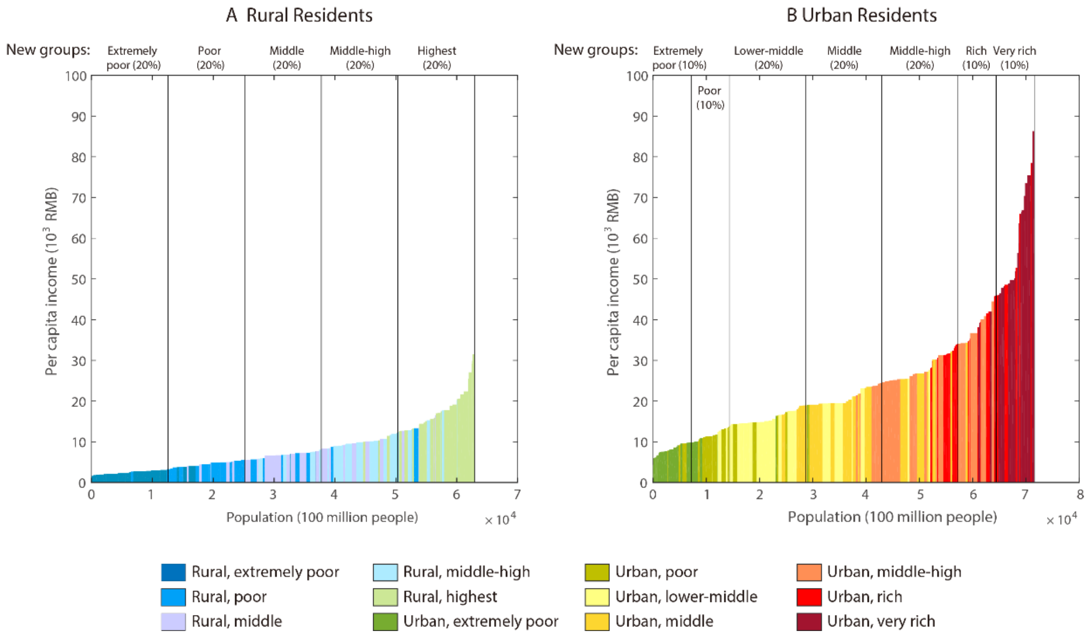

Supplementary Figure 6. The rearrange of income groups in (A) rural and (B) urban China from 30 provinces. The colors of the bars represent the original groups in each provinces. The new national groups are marked at the top of each panel. Source data are provided as a Source Data file.

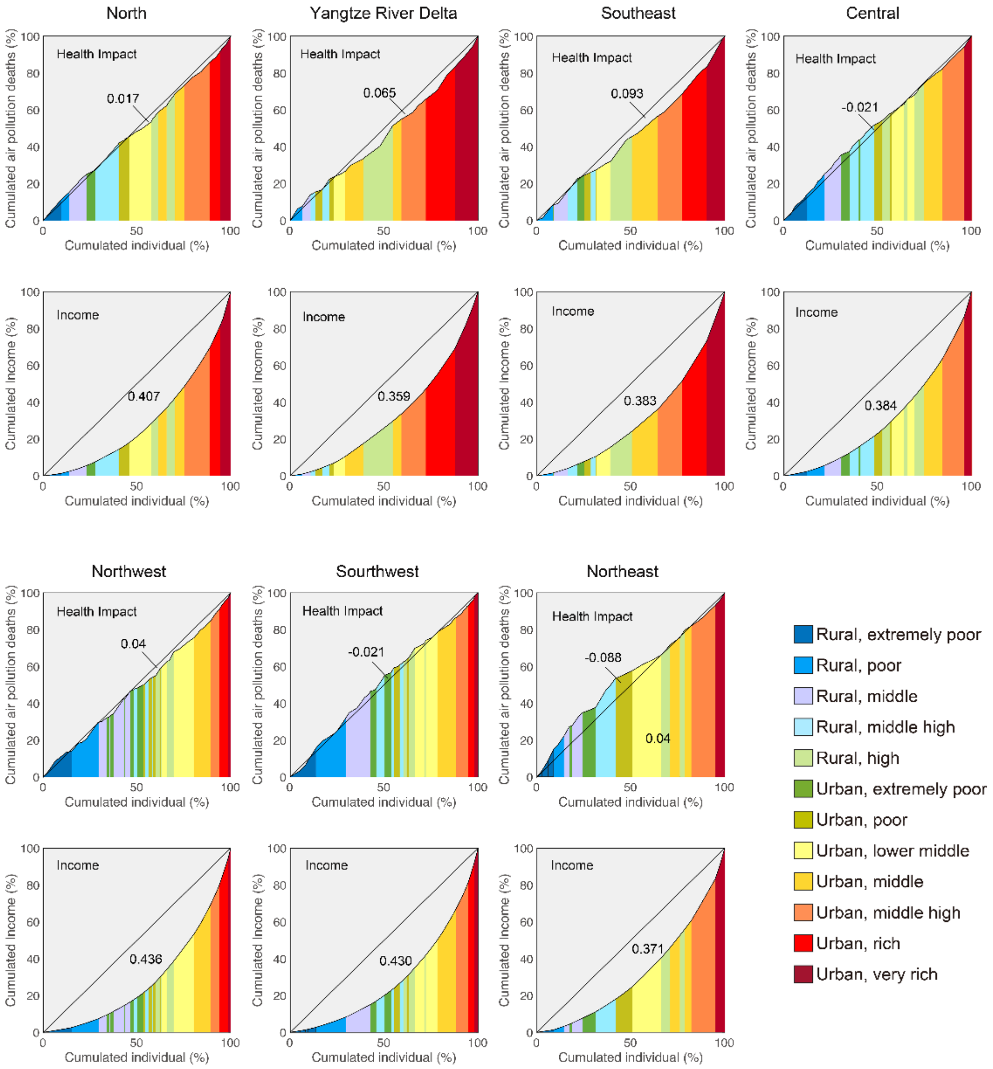

Supplementary Figure 7. Lorenz curves for household consumption related air pollution deaths caused (upper figure in each panel) and income earned (lower figure in each panel) for households in seven regions of China. Source data are provided as a Source Data file.

Supplementary Tables

Supplementary Table 1. Household consumption related premature deaths, population size, and income per capita across income groups in China.

	Population (Million people)	Household income per capita (CNY)	Consumption related deaths (10^3 persons)	Consumption related deaths per capita (10^{-4} persons)
**China, total**	**1345 (100%)**	**16950**	**482.4 (328.2~636.7) (100%)**	**3.6 (2.4~4.7)**
**Urban, total**	**716 (53%)**	**24638**	**218.2 (148.4~287.9) (45.2%)**	**3.0 (2.1~4.0)**
Very rich	72 (5.3%)	58967	38.4 (27.0~49.8) (8.0%)	5.4 (3.8~7.0)
Rich	72 (5.3%)	38383	28.5 (18.7~38.4) (5.9%)	4.0 (2.6~5.4)
Middle high	143 (10.6%)	27880	50.8 (36.2~65.5) (10.5%)	3.6 (2.5~4.6)
Middle	143 (10.6%)	20837	41.1 (29.9~52.2) (8.5%)	2.9 (2.1~3.6)
Lower middle	143 (10.6%)	15865	33.6 (24.2~43.1) (7.0%)	2.4 (1.7~3.0)
Poor	72 (5.3%)	11580	14.1 (10.3~18.0) (2.9%)	2.0 (1.4~2.5)
Extremely poor	72 (5.3%)	8288	11.5 (8.2~14.8) (2.4%)	1.6 (1.1~2.1)
**Rural, total**	**629 (47%)**	**8207**	**264.2 (179.8~348.7 (54.8%)**	**4.2 (2.9~5.5)**
Highest	126 (9.4%)	17414	57.7 (45.2~70.3) (12.0%)	4.6 (3.6~5.6)
Middle high	126 (9.4%)	9887	55.4 (42.1~68.7) (11.5%)	4.4 (3.3~5.5)
Middle	126 (9.4%)	6731	50.9 (39.3~62.5) (10.6%)	4.0 (3.1~5.0)
Poor	126 (9.4%)	4503	47.5 (35.8~59.3) (9.8%)	3.8 (2.8~4.7)
Extremely poor	126 (9.4%)	2502	52.7 (38.1~67.3) (10.9%)	4.2 (3.0~5.3)

Supplementary Table 2. Region Definitions.

Region	Provinces/municipalities included in each region
**North**	Beijing, Tianjin, Hebei and Shandong
**Yangtze River Delta**	Shanghai, Jiangsu and Zhejiang
**Southeast**	Fujian, Guangdong and Hainan
**Central**	Henan, Anhui, Hubei, Hunan and Jiangxi
**Northwest**	Shaanxi, Shanxi, Gansu, Qinghai, Ningxia, Xinjiang and Inner Mongolia
**Southwest**	Sichuan, Chongqing, Guizhou, Yunnan and Guangxi
**Northeast**	Liaoning, Jilin and Heilongjiang

Supplementary Table 3. Sector mapping between sectors in MRIO and those in Households consumption statistic data.

ID	Sectors in MRIO	Sectors in household consumption data
1	Agriculture	Grain; Starches and Tubers; Beans and Bean Products; Meats, Poultry, Egg, Aquatic Products and Related Products; Vegetables; Dried and Fresh Melons and Fruits
2	Coal mining	Residence-Water, Electricity and Fuels
3	Petroleum and gas	Residence-Water, Electricity and Fuels
4	Metal mining	Residence-House
5	Nonmetal mining	Residence-House
6	Food processing and tobaccos	Oil and Fats; Condiments; Sugar, Tobacco, Liquor and Beverages; Cake, Milk and Its Products; Other Food
7	Textile	Clothing Materials; Bed Articles
8	Clothing, leather, fur, etc.	Garments; Shoes; Other Clothing
9	Wood processing and furnishing	Furniture; Furniture Materials
10	Paper making, printing, stationery, etc.	Cultural and Recreational Articles; Teaching material
11	Petroleum refining, coking, etc.	Transport equipment fuels and parts
12	Chemical industry	Medicine; Health Products
13	Nonmetal products	Residence-House
14	Metallurgy	Residence-House
15	Metal products	Household Appliances
16	General and specialist machinery	Household Appliances
17	Transport equipment	Transportation Facility
18	Electrical equipment	Household Appliances
19	Electronic equipment	Communication Facility
20	Instrument and meter	Medical Appliances; Health Care Appliances
21	Other manufacturing	Room Decorations; Household Articles for Daily Use; Miscellaneous Goods
22	Electricity and hot water production and supply	Residence-Water, Electricity and Fuels
23	Gas and water production and supply	Residence-Water, Electricity and Fuels
24	Construction	Residence-House
25	Transport and storage	Traffic Fare
26	Wholesale and retailing	Expenditure on Food (except Dining Out), clothing, Household Appliances, Transport and Communications Facility, Cultural and Recreational Articles, and Miscellaneous Goods

27	Hotel and restaurant	Dining Out
28	Leasing and commercial services	Miscellaneous Services
29	Scientific research	Education Tuition
30	Other services	Food Processing Service Fees; Tailoring and Laundering Services; Housing Services; Household Services; Health Care Services; Transport equipment using and upkeep fare; Communication Services; Expenditure of Culture and Recreation; Education Tuition; Miscellaneous Services

Supplementary Table 4. The coefficient of variation (CV) of the estimated direct and indirect emissions in each income group under different sensitivity scenarios.

	Species	R1	R2	R3	R4	R5	U1	U2	U3	U4	U5	U6	U7
Indirect	NH_3	0.07	0.03	0.01	0.02	0.05	0.03	0.02	0.01	0.00	0.01	0.01	0.02
	SO_2	0.08	0.03	0.02	0.02	0.04	0.03	0.02	0.01	0.01	0.01	0.01	0.02
	NO_x	0.08	0.03	0.02	0.02	0.04	0.03	0.02	0.01	0.01	0.01	0.01	0.02
	BC	0.08	0.03	0.02	0.02	0.04	0.04	0.02	0.02	0.01	0.02	0.01	0.03
	OC	0.08	0.03	0.02	0.02	0.04	0.04	0.02	0.02	0.01	0.02	0.01	0.03
	$PM_{2.5}$	0.09	0.03	0.02	0.02	0.04	0.03	0.02	0.02	0.01	0.01	0.01	0.02
Direct	NH_3	0.00	0.00	0.00	0.00	0.00	0.22	0.15	0.10	0.05	0.05	0.05	0.06
	SO_2	0.13	0.10	0.04	0.07	0.10	0.04	0.03	0.01	0.01	0.02	0.02	0.02
	NO_x	0.02	0.01	0.01	0.01	0.02	0.05	0.05	0.03	0.02	0.02	0.03	0.04
	BC	0.02	0.02	0.01	0.02	0.03	0.04	0.03	0.02	0.01	0.02	0.02	0.03
	OC	0.01	0.01	0.00	0.01	0.01	0.04	0.03	0.02	0.01	0.02	0.02	0.03
	$PM_{2.5}$	0.01	0.01	0.01	0.01	0.02	0.04	0.03	0.02	0.01	0.02	0.02	0.03

Supplementary Table 5. Parameters and death incidences used in IER model.

	IHD	Stroke	COPD	LC	Source
$\boldsymbol{\alpha}$	0.843	1.01	18.3	159	Lee et al., 2015
$\boldsymbol{\gamma}$	0.0724	0.0164	0.000932	0.000119	Lee et al., 2015
$\boldsymbol{\delta}$	0.544	1.14	0.682	0.735	Lee et al., 2015
$\boldsymbol{C_0}$	6.96	8.38	7.17	7.24	Lee et al., 2015
$\boldsymbol{B}$	0.000707	0.00129	0.000696	0.000383	Forouzanfar et al., 2015

Supplementary Discussion

Uncertainties and limitations

The estimation of premature deaths associated with household consumption related atmospheric pollutant emissions are subject to a number of uncertainties, due to the limitation and assumption inherit of the models used in each step. The uncertainty ranges (95% confidence interval) in different steps of our analysis and the overall uncertainties are discussed below.

First, there are uncertainties in the air pollutant emission inventory, due to the incomplete knowledge on activity level, combustion/production technology and emission factors. Zhao et al.[1] estimate that the uncertainties of China's anthropogenic SO_2, NO_x, $PM_{2.5}$, BC, and OC emissions are −14%~13%, −13%~37%,−17%~54%, −25%~136% and −40%~121%, respectively. The MEIC model used in this study have similar uncertainty ranges, with lower uncertainties associated with SO_2 and NO_x than BC and OC. In addition, the MEIC model has been widely used and proved reliable in chemical transport simulations when validated against surface and satellite observations[2-4].

Second, using MRIO analysis and statistical data to allocate production-based emissions to direct and indirect household consumption in different income groups introduces additional uncertainties. Uncertainty in MRIO model is from its data source and data manipulation process, such as sector and region aggregation, data harmonization[5,6]. By using Monte Carlo simulation, Lin et al.[7] estimates that the uncertainties of China export-related pollutant emissions is ±50% and the input-output model contributed to ~10% of the total errors. We consider a 10% of uncertainty to represent the errors brought by MRIO analysis. The uncertainties associated with the statistical data mainly come from the missing of income groups and expenditure patterns data in some provinces, which have been discussed in S3. We use the CV values of each income groups as the uncertainty estimates of the statistical data, as shown in Supplementary Table 4.

Third, errors in the total premature deaths related to $PM_{2.5}$ pollution come from satellite-based $PM_{2.5}$ estimates and health impact models. The $PM_{2.5}$ concentration used here has been calibrated by satellite-based and surface observations, so its uncertainty is relative small, about ±5 % on average according to GBD 2013[8]. For the IER model, it is fit by incorporating information on risk due to various emission sources (ambient air pollution, first- and second- hand tobacco smoking, and household indoor air pollution) covering a wide range of $PM_{2.5}$ concentration, but with limited information on actual exposure to ambient $PM_{2.5}$ at higher and lower concentrations[9]. Moreover, the IER function is limited to the assumptions that each health endpoint is independent of exposure period, $PM_{2.5}$ composition and toxicity for particles from different sources[9]. It is also notable that the IER function has been updated by Cohen et al.[10], yielding about 35% higher mortality estimates compared to previous work. The uncertainty associated with IER model is quantified by 1,000 simulations using 1,000 sets of parameters provided by Burnett et al. [9]. Uncertainty in the $PM_{2.5}$ data is minor compared to that of IER model and is ignored in the simulations.

Fourth, uncertainties in linking the total premature deaths into each income group also arise from the simulation of GEOS-Chem and its adjoint. The model simulations can contain a lot of uncertainties due to the uncertainty in emission inputs and the model's imperfect representation of chemical and physical process, such as the chemical conversion and physical transport and transform. For GEOS-Chem and its adjoint, the uncertainties come from both the forward simulation

and the backward response process. For the forward process, we conduct a comparison between the modeled and the satellite-derived $PM_{2.5}$ concentration among seven regions in China (Supplementary Figure 3), and the R range from 0.28 to 0.95. For the backward process, the calculation is more complex due to its frequent integration with the forward process. Moreover, the source attribution using adjoint sensitives implicitly neglect the nonlinear response of $PM_{2.5}$ to emissions changes. Due to the complex interaction process, there are very few statistical quantification for the uncertainties in GEOS-Chem adjoint simulation so far. We used 30% to represent its uncertainty in our study.

The overall uncertainties involved in the household consumption related premature deaths attributable to $PM_{2.5}$ in different income groups are determined by uncertainties in total $PM_{2.5}$ related death calculated by IER functions and the fractional contribution of each income group calculated using GEOS-Chem adjoint model, emission inventory, MRIO model and the statistical data. Uncertainty of total $PM_{2.5}$ related death follows the distribution generated by 1,000 sets of IER parameters. Uncertainties in the fractional contributions are addition in quadrature of errors in GEOS-Chem adjoint model, emissions inventory, MRIO model and the statistical data. Finally, the overall uncertainties are derived from aggregations of errors above. We present the 95% confidence interval of the overall uncertainties in our study.

Supplementary References

1 Zhao, Y., Nielsen, C. P., Lei, Y., McElroy, M. B. & Hao, J. Quantifying the uncertainties of a bottom-up emission inventory of anthropogenic atmospheric pollutants in China. *Atmos. Chem. Phys.* **11**, 2295-2308 (2011).

2 Li, X. *et al.* Source contributions of urban $PM_{2.5}$ in the Beijing–Tianjin–Hebei region: Changes between 2006 and 2013 and relative impacts of emissions and meteorology. *Atmos. Environ.* **123, Part A**, 229-239 (2015).

3 Zheng, B. *et al.* Heterogeneous chemistry: a mechanism missing in current models to explain secondary inorganic aerosol formation during the January 2013 haze episode in North China. *Atmos. Chem. Phys.* **15**, 2031-2049 (2015).

4 Hu, J., Chen, J., Ying, Q. & Zhang, H. One-year simulation of ozone and particulate matter in China using WRF/CMAQ modeling system. *Atmos. Chem. Phys.* **16**, 10333-10350 (2016).

5 Wiedmann, T., Wilting, H. C., Lenzen, M., Lutter, S. & Palm, V. Quo Vadis MRIO? Methodological, data and institutional requirements for multi-region input–output analysis. *Ecol. Econ.* **70**, 1937-1945 (2011).

6 Tukker, A. & Dietzenbacher, E. Global multiregional input-output frameworks: an introduction and outlook. *Econ. Syst. Res.* **25**, 1-19, (2013).

7 Lin, J. *et al.* China's international trade and air pollution in the United States. *Proc. Natl. Acad. Sci. USA* **111**, 1736-1741 (2014).

8 Brauer, M. *et al.* Ambient Air Pollution Exposure Estimation for the Global Burden of Disease 2013. *Environ. Sci. Technol.* **50**, 79-88 (2016).

9 Burnett, R. T. *et al.* An Integrated Risk Function for Estimating the Global Burden of Disease Attributable to Ambient Fine Particulate Matter Exposure. *Environ. Health Perspect.* **122**, 397-403 (2014).

10 Cohen, A. J. *et al.* Estimates and 25-year trends of the global burden of disease attributable to ambient air pollution: an analysis of data from the Global Burden of Diseases Study 2015. *The Lancet* **389**, 1907-1918 (2017).
